# Trapped Between Two Pandemics: Domestic Violence Cases Under COVID-19 Pandemic Lockdown: A Scoping Review

**DOI:** 10.1177/0272684X211022121

**Published:** 2021-06-08

**Authors:** Endurance Uzobo, Aboluwaji D Ayinmoro

**Affiliations:** 1 Department of Sociology, Niger Delta University, Wilberforce Island, Nigeria; 2 Department of Sociology, University of Ibadan, Ibadan, Nigeria

**Keywords:** domestic violence, COVID-19, Africa, gender-based violence, lockdown

## Abstract

**Background:**

As it is common with the most devastating events in the world, women always seem to be at the most disadvantage position. This situation manifested during the period of COVID-19 lockdown throughout the world and Africa in particular. The purpose of this study is to explore Domestic Violence (DV) cases in African during the COVID-19 lockdown.

**Methods:**

Data for this study were gleaned from an electronic literature search using various databases PubMed and BioMed Central, Web of Science, etc. Key search words were gender DV during and after COVID-19. A total of 68 records were identified during the search. However, only 46 of these sources met the inclusion criteria.

**Results:**

From the review done in selected African countries which include Egypt, South Africa, Kenya, Nigeria, Ghana and Zimbabwe; it was discovered that COVID-19 lockdown across these countries worsens the already existing cases of DV. The study also noted that generally, the response of the government has been very poor in terms of dealing with DV cases in the period of COVID-19 lockdown.

**Conclusion:**

The study concluded that despite the failures of government in tackling the DV pandemics, NGOs have been very active in championing the cause of those violated while also trying to provide succour to victims. Thus, the study recommended that countries in Africa need to join international initiatives in prioritising DV cases while trying to deal with the virus itself. Thus, one disease should not be traded for another.

Understanding crises through a social relations lens gives a clearer picture of the social structure of societies, organisations, households, and other intimate relationships within which catastrophic events emanates. Hence, paying attention to gender relations during disaster gives us a better understanding of how to critically comprehend power dynamics in pandemic situations.^1^ This is because as evidence has shown from various studies and observations in times of disastrous events, women may experience a re-emphasis of (i) traditional and lower household status (ii) worsening condition.^1–3^

However, while intimate partner violence (IPV), domestic violence (DV) and Gender-based violence (GBV) are often confused and used interchangeably, it is important that we briefly outline their differences. IPV is usually used to refer to any behaviour that occurs within an intimate relationship between partners or ex-partners (either married or cohabiting) which can lead to physical, psychological or sexual harm to those involved in the intimate relationship.^4^ The US National Institutes of Mental Health Committee on Family Violence however proposed a broader description of this concept when it defined it as “acts that are physically and emotionally harmful or that carry the potential to cause physical harm and may also include sexual coercion or assaults, physical intimidations, threats to kill or harm, restriction of normal activities or freedom and denial of access to resources”.^5^ Domestic Violence (DV), on the other hand, was also defined by WHO to includes all acts of aggression which might be physical, psychological, sexual and economical in nature which occur within a domestic unit such as families and between intimate partners. Thus, DV could be against a woman or child/adolescent by an intimate male partner/cohabiting partner, parents, siblings, family relatives, or anyone well known to the family.^6^ In other words, the term DV is a broader concept than IPV. Furthermore, GBV which is a broader concept than IPV and DV refers to violence directed at a person because of their gender. It is usually used to generally capture any form of violence that is rooted in exploiting unequal power relationships between genders. Just like IPV and DV, GBV could also occur in the following form; physical, sexual, psychological and economic harm (European Commission, No date). Given the fact that IPV is a subset of DV, while GBV is broader, in this study we shall be using the three concepts interchangeably.

Gender-based violence (GBV) against women and girls has been identified as one of the most visible manifestations of gender inequalities. Thus, studies have stated that DV increases during crises situations and that it occurs in all countries of the world irrespective of the stages of development.^7,8^ A typical example is the 2010 disaster in East Japan where women and girls were required in the evacuation centres to prepare meals.^3^ Similarly in Haiti, women living in internally displaced persons (IDPs) camps after the 2010 earthquake coped with male-dominated committees who controlled aid distribution, hence, some women were forced to negotiate for relief materials through sexual favours.^9^ Similar scenarios in IDP camps in North-east Nigeria has also been reported.^10^ Nevertheless, due to societal pressure, the capacities of victims to report abuse is oftentimes impeded.^3^

Also, following the Hurricane Katrina in the United States in 2005, the rape rate among displaced women was reported to be 53.6 times more than the highest baseline rate for Mississippi in 2004; with intimate partner rape 16 times higher than the US yearly rate.^11^ More so, in Uganda, a need assessment survey in Karamoja showed that violent practices such as courtship rape, DV, child marriage, and female genital cutting, increased during droughts.^12^

In the war-torn region of the middle-east, almost a decade after the crisis broke out, reports have shown that violence against women and girls have continued unabated. In Syria, Yemen, Iraq, Libya and Somalia different forms of violence perpetrated against women and girls such as sexual harassment, child marriage, forced marriage, domestic abuse, sexual violence and other forms of GBV have been reported right from the beginning of the crises.^13^

The above examples demonstrate how crises such as pandemics, natural/man-made related disasters and armed-conflicts aggravate skewed gender relations. It has also been shown that the dependence on familial assistance during and after a crisis may also strengthen established power dynamics, which additional remove agency and control in the hands of women.^14^ Despite the critical insights a gender analysis provide, gender-sensitive evaluations and assessments such as the collection of data on sex, age, and disability are not thoroughly conducted during and after a pandemic situation.^8,15^ This situation is still in a replay in the wake of the coronavirus pandemic in the world.

The role women and girls play during disaster and crises situations have also been found to influence the risk factor of GBV. For example, while scarcity of water in the Sahel forces women and girls to walk long distances to fetch water and fuel, there is a likelihood of their increasing exposure to the risk of harassment and sexual assault.^1,16,17^

Additionally, studies have indicated that addiction to alcohol, drugs and gambling used mostly by men during disasters as coping strategies serve as a risk factor of increased violence against women and girls.^15,18^ For instance, in an assessment of the post-Cyclone Nargis in Myanmar, findings revealed that an increase in alcohol consumption led to about a 30% increase in DV after the disaster.^19^ Given that women and girls are usually exposed to the risk of GBV during a crisis, it is hypothesised that the likelihood of women being at the receiving end of DV might emanate especially at the family level during ‘stay-home order’ of coronavirus pandemic. Hence, this study sought to explore the trends and patterns in DV in the period of COVID-19 lockdown in Africa.

## Materials and Methods

**Figure 1.**
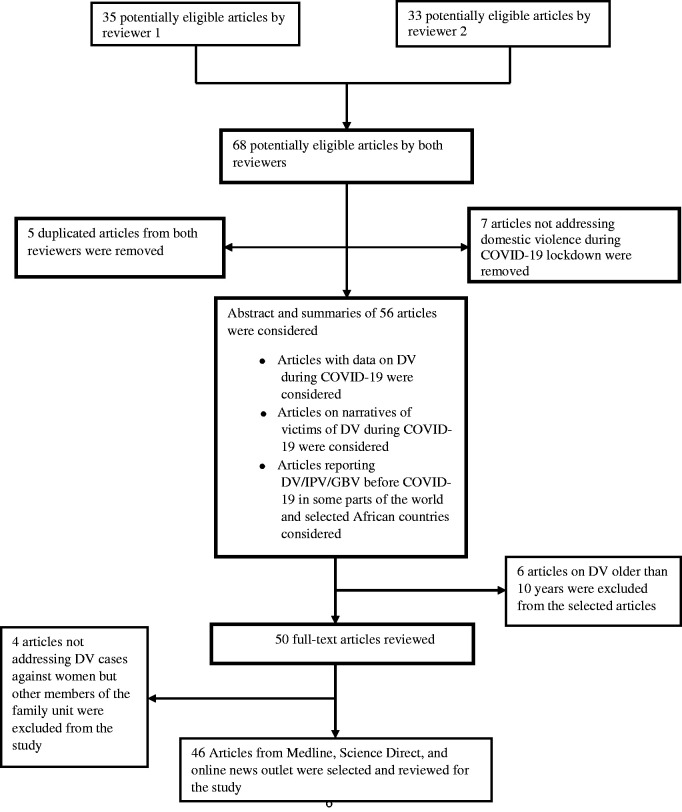
Scoping Literature Review Flow-Chart.

Since, empirical research on DV cases against women during the COVID-19 pandemic lockdown are still ongoing, this study adopted an exploratory research design to explore the situation of DV especially in Africa during the coronavirus pandemic lockdown. Given the nature of this study as a scoping review, published articles used in this study were gleaned from secondary sources searched from reputable databases. In the search for literature, this study narrowed its focus on the articles published on the subject matter between 2010 to 2020. Key search words used in the search were DV cases before COVID-19 lockdown, DV cases after COVID-19 lockdown in the world and Africa. IPV during COVID-19 lockdown, GBV during COVID-19 lockdown, DV during crisis situations, etc. The database where secondary data were sourced from include; PubMed and BioMed Central, Web of Science, Access World News, PsychInfo and Africannews. The two authors (EU and ADA) independently searched and reviewed articles addressing the keywords. This process yielded a total of 68 articles made up of systematic reviewed studies, commentaries and newspapers publications on the subject matter. After this process, articles which did not properly address the main objectives of the study were excluded from the study. After the two authors reached a mutual decision on selected articles, a total of 46 successful articles were reviewed for the study (see Figure 1). Thus, authors considered only articles that contained information addressing DV cases against women before and after the coronavirus pandemic. Also, in determining the eligibility criteria for the selection of materials used for the study, priority was given to materials that demonstrate DV in different African countries especially during the period of the lockdown occasioned by the outbreak of the coronavirus.

## Findings

### Some Cases of DV During COVID-19 Lockdown Around the World

Though it is often said that ‘absence makes the heart grow fonder’, the opposite might be the case if too much time is spent in close spaces as humans in modern times value and need personal space. Lack of personal space is said to increase the level of interpersonal pressures which eventually leads to conflict.^20^ This position is also buttressed by the Chinese proverb: “it is the distance that produces beauty and comfort”. Still taking the same stance, Marianne Hester- a Bristol University sociologist of an abusive relationship,^21^ stated that the more time families spend together in events such as Christmas and summer vacations, DV rise is probable. More so, Jenny Beck - a family Lawyer,^20^ added that women will die if they are imprisoned in their homes. This has been supported by other studies which state that domestic abusers are more likely to murder their partners and others in the wake of personal crises.

There is no doubt that the outbreak of diseases and infirmities tends to feel the impact differently. Thus, is expected that all vulnerable groups will experience the impact of the COVID-19 outbreaks differently. It is on this note that the UNFPA^2^ stated that about 48 million women and girls, including 4 million pregnant women, require humanitarian assistance and protection in 2020 as a result of the COVID-19 outbreak.

Statistics on DV cases against women prior to the COVID-19 lockdown though high, was made worse by the lockdown down policy of most national government. For instance, the WHO in 2017 indicated a global estimate of about 1 in 3 (35%) of women worldwide who have experienced either physical and/or sexual IPV or non-partner sexual violence in their lifetime. The WHO further added that most of the DV cases are IPV with about 38% of women who were murdered being committed by a male intimate partner”.^22^ A Meta-analysis of prevalence of DV cases in Arab countries revealed a 73.3% estimate of lifetime exposure to any type of IPV, 35.6% physical IPV, 22% sexual IPV and 49.8% emotional/psychological IPV 49·8%.^23^ Additionally, a 2019 multilevel study in Nigeria indicated that almost one in four women have experienced IPV (23.6%), while one in five (20%) have reportedly experienced any form of IPV. Of the three forms of violence in the study, it was discovered that emotional violence was highest (18%).^24^ From the two studies above, it could be seen that psychological/emotional violence seems to be the most prevalence before the outbreak of the coronavirus.

While some lucky couples have rediscovered marital bliss in the wake of the coronavirus pandemic lockdown through spending more time to an intimate relationship and communication with family members and consequently boosting family harmony; it has, on the other hand, become a serious public health concern due to the risk of GBV. Journalistic reports from all parts of the world show that there are conjugal strife and crises as DV and divorce has more than tripled with a short period of about two months. Media reports from various cities in China indicated that there was an upsurge of uncouplings and DV in March 2020. Specifically, Shanghai-based online publication Sixth Tone^25,26^ reported that police in one county alone near where the pandemic began in Wuhan, received 162 reports of DV in February, which was more than tripling the 47 reported cases during the same month in 2019. A similar trend was also reported at the southern Hubei province in Jianli County. According to Wan Fei-a retired police officer,^26^ about 90% of the causes of DV between the period February in the Jianli County of China was related to the COVID-19 epidemic.

Home isolation orders present abusers with increased opportunity to inflict harm on victims who are rendered more vulnerable by reduced access to their support networks and limited options for escape from the home. Governments struggling to respond to the coronavirus epidemic have failed to respond to this spill-over effect – and to a similar crisis affecting vulnerable children – with increased services that cater to those at risk. This has left DV response centres overwhelmed by the heightened demands on their services.^27^

In Spain, Ana Bella the founder of an NGO^21^ reported that the emergency number for IPV received 18 per cent more calls in the first two weeks of lockdown than in the same period a month earlier. Still, the French police reported a nationwide spike of about 30 per cent in DV cases in just one day. In Britain, though lockdown was effected late, Avon and Somerset^21^ stated that domestic abuse cases were up by 20% in the southwest part of the country. In the United States, the National Domestic Violence Hotline^28^ reports that a growing number of callers say that their abusers are using COVID-19 as a means of further isolating victims from friends and family, threatening to throw their victims out on the street so they get sick and withholding financial resources or medical assistance.

In Lebanon, Ghida Anani – the director of the Abaad Resource centre for gender equality^27^ reported that from calls received by the centre so far, sever life-threatening DV against women at home are emerging, with an average of about two women receiving death threats from family members after showing flu-like symptoms associated with coronavirus. This she further added has led to a whole lot of mental health issues and the experience of suicidal thoughts. Besides, Lewis^29^ opined that the majority of women (60%) who contacted Lebanese women’s protection non-governmental organization KAFA’s hotline in March were doing so for the first time, reporting new incidents of physical violence or psychological abuse committed during the lockdown.

One reason attributed to the increasing cases of DV, according to Feng Yuan; a co-founder of a Beijing based non-organisation^25^ is because “lockdown brings out latent tendencies for violence that were there before but not coming out”. Again, Taub,^21^ also stated that the cases of DV during the period of COVID-19 lockdown might be as a result of a shattered support network which has made it more difficult for victims to get help. Other reasons ranging from marital irritants, finance, screen time, homework, childcare, infidelity, lack of personal space, and anxiety from quarantine have been attributed to be the cause from various newspaper reports.^21,22,29^

Apart from causing sexual harassment and violence, child marriage, forced marriage, domestic abuse, it is important to note that DV also causes women’s deaths. This has been the main problem in France during the COVID-19 home confinement. Statistics from France suggests that a French woman die every three days due to issues associated with DV.^6,30^

Although many developed countries have reported cases of DV during the period of COVID-19 lockdown, African countries have remained silent with few of her countries reporting limited cases of DV. Hence, if this issue is not also addressed while battling the coronavirus pandemic, African countries might have a new public and mental health crises to deal with in the future. Hence, addressing the issue of COVID-19 as a risk factor of DV alongside with the efforts of the global community to curtail the spread of the pandemic through biomedical and epidemiological frameworks will not only provide a holistic approach towards ending its negative consequences, but helps to facilitate the achievement of healthy lives and gender equality of the SDGs by 2030.

### African Scenario

The African continent has been relatively lucky concerning the outbreak of the pandemic when compare to other continents with a higher proportion of reported cases and deaths emanating from the disease. However, it is more worrisome that the continent that feels the latent effects of the lockdown occasioned by the pandemic cannot be easily assessed. For instance, the lockdown was declared in March 2020 by most countries in Africa. While, some of the countries in the continent started their lockdown with selected major cities, others declared a nation-wide lockdown.

The Nigerian government, as an example, first declared a total lockdown in three cities namely; Abuja (Federal Capial Territory), Lagos (Lagos State) and Abeokuta (Ogun State) on the 30th of March, 2020. In his move to contain the spread of coronavirus the first case was recorded in early March 2020, the South African president, Cyril Ramaphosa also announced a 21-day national lockdown on Wednesday 23rd of March, 2020 but to take effect on Thursday March 26. Similarly, Egypt on her part imposed a nationwide lockdown, including a full night-time curfew, on Wednesday 23rd of March, 2020 to combat the spread of the the pandemic in the country. Those who violates lockdown measures were expected to face hefty legal sanctions ranging from a fine of up to 4000 Egyptian pounds, including a possibility of prison sentence. Due to the rise in the spread of the pandemic in most African countries, the initial declarations of the lockdown for two to three weeks in March 2020 were later extended to July and September, 2020.

**Figure 2.**
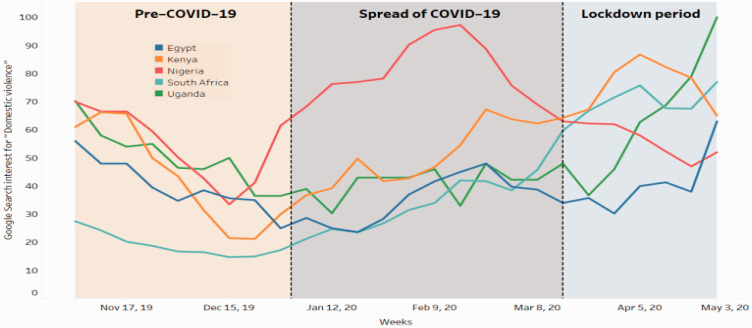
Google Searches for ‘Domestic Violence Help’ in Selected African Countries Since COVID-19.^28^

While the lockdown and stay-at-home order including the use of face mask, washing of hands for at least one minute, social and physical distancing were adopted by most African countries to curb the spread of the virus, there were reports on the incidences of DV against women. Take for example, Google searches on DV in Africa indicate a spike in the number of people seeking help dealing with DV and sexual harassment since the start of COVID-19 (see Figure 2). In this section, we explore some of the reported cases of DV in Africa.

Common risk factors of DV identified during the coronavirus pandemic lockdown include; The lowered economic activities especially for women, as they are more likely to be poor in period of crises, too much screen time, housework and child care, food insecurity due to the loss of jobs and incomes, and an increase in the use of alcohol and/or substance especially by the men.^25^

### Egypt

According to the UNDP Gender and Justice report on Egypt,^31^ no law explicitly refers to domestic violence in Egyptian document. Thus, while some domestic violence offences may be punishable under the Penal Code and Law No. 6 of 1998, it is only if it is considered to be beyond “the accepted limits of discipline decided by the judge” and “if the injuries are apparent” when filing the complaint at the police station. In contrast, Article 60, for instance, can still be used by the perpetrator to be pardoned if he acted in “good faith”, which is regarded as “the husband’s right to discipline his wife”.^31^

Hence, like many other African and Middle-eastern countries, DV is still tolerated by many Egyptians. Therefore, it is not a coincidence that Egypt was ranked second in the world after Afghanistan in terms of sexual harassment.^32^ A study by the Ministry of Health,^4^ stated that nearly half of all women surveyed stated that they had experienced some form of DV in Egypt. The Survivors of DV further interviewed by Amnesty International described brutal physical and psychological abuse by spouses as the most prevalent form of violence witnessed, saying that their spouses had beaten, whipped and burned them and in some cases locked them up inside the house against their will.^7^ In a similar vein, a study by Egypt's National Council of Women (ENCW)^33^ stated that about 1.5 million women reported being subjected to DV each year. A further breakdown of this figure by ENCW indicated that approximately 4,000 women are being abused on a daily bases in Egypt, even though there are a whole lot of unreported cases of DV.

A study by UN women in 2013 (32) on “Ways and Methods to Eliminate Sexual Harassment in Egypt” further buttressed this point when it noted that over 99.3% of Egyptian girls and women surveyed reported experiencing some form of sexual harassment in their lifetime. The same study also noted that 82.6 per cent of the total female respondents did not feel safe or secure in the street. The number increased to about 86.5 per cent concerning safety and security in public transportation.

With regards to DV emanating from COVID-19 lockdown, Abdulaal^31^ observed that the suspension of court trials across the country has led to an increase in the mumber of partners who were perpetrators of dastardly act of DV against their spouses. Consequently, a report from Egyptian Centre for Women’s Rights (ECWR) have found out that since the beginning of the shutdown in the country, there has been a surge in family conflict and cases of violence, representing 43 per cent of the total number of 1146 cases received, with over 70 per cent of the complaints received by women.^31^ Another report discovers a 33% increase in family problems, 19% increase in violence between family members, and 11% of wives subjected to violence from their husbands.^31^ Dependence on husbands and children is often a factor reported by women to be the cause of DV during the period of lockdown, which reveal the unequal nature of the relationship that characterizes abuses in the households.

### South Africa

Before the outbreak of the COVID-19 pandemic, the reported cases of GBV in South Africa were among the highest in the world. For instance, reports have it that in South African, a woman is murdered every three hours on the average, with many assaulted and raped before being killed.^34^ This trend had already ignited protests in most parts of the country. This drew the attention of the South African government in September 2019 which recognise the dire state of women within the country, thereby declaring GBV and femicide as a national crisis.

In South Africa, where COVID-19 cases have been concentrated, reports have it that during the period of the COVID-19 lockdown, about 148 people have been arrested and charged with crimes related to GBV, and over 2,000 complaints of GBV were made to the South African Police Service in the first seven days of the lockdown.^35^ Additionally, the GBV National Command Centre, that operates a national call centre facility reported having received about 12,000 calls on DV since the implementation of the lockdown. There were also reports of the rape of women in temporary camps for homeless people erected as part of a COVID-19 response.^36^

Nevertheless, the South African government is one of the very few countries in Africa to enforce exceptionally strict policies to curb DV during the period of lockdown. This was done as the government prohibited the sale of cigarettes and alcohol, which have been identified as a major catalyst for DV as well as immune system suppressants.

### Kenya

Just like South Africa, GBV against women and girls across Kenya is also very high and has become a daily reality. For instance, a report has it that 45 per cent of women and girls aged fifteen to forty-nine have experienced physical violence and another 14 per cent have reported experiencing sexual violence.^37^ Others have even added that these statistics might even be lower than the actual incident of GBV against women given the fact that most sexual violence against women is under-reported because of the stigma attached to it.

In Kenya, the National Council on Administration of Justice reported a spike in sexual offences during the coronavirus pandemic lockdown, and has identified the primary perpetrators as “close relatives, guardians, and/or persons living with the victims”.^35^ According to Mutavati and Zaman,^37^ a third of all crimes reported since COVID-19 pandemic started in Kenya were related to sexual violence. Mutavati and Zaman^37^ further reported that the rise in DV during this period arose as a result of financial hardship due to restriction of movement and curfew affected the sources livelihoods, especially for those working in the informal sector. Thus, confinement at home under heightened levels of stress, uncertainty and fear produced stressful environments that precipitate DV.

### Nigeria

According to the 2018 Nigeria Demographic and Health Survey (NDHS), 31% of women age 15–49, have experienced physical violence, 9% have experienced sexual violence and 6% have experienced physical violence during pregnancy. This figure was lower for the 2013 NDHS which was put at 25%.^38^ There is no doubt that with the lockdown across the 36 states and Federal Capital Territory occasioned by the outbreak of the coronavirus, this figure will rise drastically.

Domestic violence reports from Nigeria have indicated that rape and sexual violence increased during the months of the lockdown in most states. A survey conducted by Partners West Africa Nigeria (PWAN) in three northern states of Nigeria **(**FCT, Borno and Kano states) revealed that there has been an increase in the rate of reported cases of Sexual and Gender-Based Violence (SGBV). In the words of the Special Adviser to the Chairman of Abuja Municipal Area Council (AMAC), and representative of the social welfare unit:^39^

I am currently handling a case of rape… It has been transferred to force CID for prosecution. The lockdown has lowered economic activities for everyone but women are feeling the impact more because the larger percentage of people in poverty are women. Most women rely on daily income to feed their families; women are forced to be on lockdown with their abuser because of no movement and no available alternatives causing them to endure violence.

In Kano, the report stated that a respondent alleged that a friend of hers was raped; the case was reported to the police, and the perpetrator arrested. In Lagos, the Domestic and Sexual Violence Response Team (DSVRT) the daily reported cases of domestic and sexual abuse increased by almost 50% from the start of the Coronavirus lockdown compared to just approximately 8 cases of domestic abuse previously reported before the lockdown.^39^

### Ghana

A 2016 national survey on DV in Ghana indicated that approximately 27.7 per cent of women in Ghana had experienced at least one form of DV (physical, sexual, economic, social and psychological) within last 12 months before the survey. Also, the survey estimated that 23.1 per cent of women found wife-beating acceptable by them. Furthermore, the study revealed that only an estimated number of about 9 per cent of women first report DV cases to the police.^40^

Though no official statistics are showing the incidence of DV in Ghana under COVID-19 lockdown, reports from some quarters have indicated that DV cases will likely increase during the coronavirus lockdown in Ghana. However, according to Addadzi-Koom,^41^ only a few cases are likely to be reported, given the fact that in most cases, the patriarchal nature of the Ghanaian society overlooks DV, thereby making victims less likely to see their situation as an emergency case.

### Zimbabwe

Data from Zimbabwe National Statistics Office reveals that DV especially sexual assault has continued to be on the rise in Zimbabwe. For instance, according to a survey data from 2010 to 2016, there was a 42 per cent increase in rape cases, with an estimated 21 women raped daily, even though the majority of cases are not reported (especially sexual violence) [42]. Also, the survey revealed that about 78 per cent of women indicated that they experienced DV at the hands of their husband or partner. Additionally, according to UNICEF,^42^ one in every three girls in Zimbabwe had experienced sexual violence before the age of 18.

Though Zimbabwe has one of the lowest reported cases of the COVID-19 pandemic, reports during the COVID-19 lockdown in Zimbabwe indicated that there was a surge DV within the first two weeks of the lockdown alone. Between March 30 and April 9, the Musasa Project (a member of the Peacebuilding Network in Zimbabwe which tracks and monitors conflict) reported that it received about 764 GBV cases,^43^ compared to an average of 500 it used to receive per month. Thus, Ms Ruvimbo Mushavi (Communications Officer of the Spotlight Initiative to end violence against women and girls) in her words stated that:

The levels of sexual and gender-based violence can be expected to spike in Zimbabwe as households are placed under the increased strains that come from concerns of health, psycosocial (sic), and income, and many women and girls are under lockdown with their abusers.^44^

Individuals who have not gone through any form of DV often wonder why women in abusive relationships still stay. In African setting, leaving an abusive relationship is not as easy as it is in other developed climes. Apart from the cultural and religious stigma attached to abandoning one’s home as a result of DV, for many victims, they cannot leave based on the significant mental, emotional, physical, and financial investment they have put into building and retaining the relationship.^45^ Other reasons that have been advanced on why women remain in an abusive relationship include; (i) The Fear of uncertainties e.g. fear of not finding another spouse (ii) Financial Sustainability (iii) Self-identity- The African societal expectations is that a women’s self-worth is dependent upon keeping a man and bearing his name (iv) Maternal bond with children (v) Family dignity- most women remain in abusive relationship to supposedly preserve the dignity and pride of their families (vi) The shame and stigma attached to women from broken marriages in Africa is usually very high (vii) Religious Reasons – most religious institutions and body in Africa prohibits divorce or separation.^45^ While any of the above reasons might have prevented woman from leaving their violent partners, it is also important to note that during the lockdown, there would have been nowhere for the women to run to.

### Government Response to DV Under COVID-10 Lockdown in Africa

Government responses to DV cases in Africa has been very slow compared to other continents that set up modalities to deal with this situation within the first one month of the pandemic. Nevertheless, few countries in Africa have made certain efforts to curb the situation. In South Africa, the government prohibited the sales of certain drugs and alcohol which have been identified as a major driver of DV. The South African government also ordered courts to try DV cases open during the period of the lockdown.

In a bid to curb DV during the lockdown occasioned by the coronavirus, the Moroccan government took a bold step by joining an international initiative that seeks to counter DV across the country and worldwide during the COVID-19 lockdown. Morocco took the initiative along with the EU and a group of countries to support the UN’s goal for “peace at home, in households, around the world” against DV during confinement.^46^ The initiative was jointly conceived by the UN, Morocco, the EU, and other countries like Argentina, New Zealand, Mongolia, Turkey, and Namibia. In the declaration, the signatories to the initiatives vow to position the “prevention and remedy” of DV as a key national and global response to the coronavirus pandemic.^46^

Though the efforts made by the government in African countries are minimal, nevertheless, non-governmental organisations in almost all African countries have been alive in the fight against DV during the period of the COVID-19 lockdown. For instance, in Morocco, Mobilising for Rights Associations (MRA) has assembled online emergency resources for victims of DV during the coronavirus-induced lockdown.

In Nigeria, the Lagos State Domestic and Sexual Violence Response Team (LSDSVRT) has also set up emergency lines to call to report cases of DV. The greatest support in Africa and the world at large has been from the United Nation. For instance, the United Nations Trust Fund to End Violence against Women has established a COVID-19 Funding Window, with aim supporting existing civil society organisations and also fund initiatives specifically designed to support women and girls who have experienced violence within the context of the pandemic in Africa and across the world.

### The Way Forward

Studies have indicated that victims and survivors of DV are at higher risk of health-related problems such as sexually transmitted infections, gynaecological dysfunction, chronic pain, and Post-Traumatic Stress Disorder (PTSD). These latent health-related consequences often continue long after the pandemic and abuse have ended.^36^ Thus, to prevent these latent effects of the COVID-19 lockdown, there is a need for several measures to be put in place by all stakeholders in GBV, particuary domestic violence through community sensitisation to increase the level of reportage, while promoting GBV legislations. In view of this, it is recommended that:

Firstly, there is need for countries in Africa to heed the International Commission of Jurists calls in order to discharge their human right obligations to eliminate GBV during crisis or confinement situations. This follows that all governments of African countries owe their citizes the obligations to maintain human rights protection just as the pandemic was declared a serious public health emergency.

Again, there is a need for African countries to give high priority to comprehensive measures that will address DV and other forms of GBV. Hence, they must assign sufficient human and financial resources expedient to tackle wide cases of DV within the context of the COVID-19 lockdown. In other words, certain personnel could be trained to rapidly respond and handle DV cases during lockdown situations, while bringing all perpetrators of DV cases to justice.

Furthermore, just as most European and North American countries have done, there is need for African countries to empower NGOs to increase their efforts to raise awareness of the criminal nature of DV and the services available to victims during lockdowns. While empowering these NGOs to create awareness, the governments in African countries must also ensure that measures such as the provision of physical and mental healthcare services, housing and shelter services, and police and justice services have been created for victims to seek succour.

Additionally, drugs and other stimulants that are likely to increase the rate of DV must be properly monitored by drug law enforcement agencies in Africa to ensure that they are out of the markets. Though South Africa has prohibited certain drugs during the COVID-19 lockdown, the policy action to ensure that this policy is carried out to the later has not been put in place.

Finally, DV courts must be opened and the means of ensuring that access to these courts made it easier by the governments in the continents to prosecute DV perpetrators. This will send a strong signal to perpetrators of all forms of GBV that justice has not gone on holidays but readily available to protect the fundamental human rights of women and girls or any other vulnerable group of DV across the African Continent.
